# Whole Genome Sequences of *Cryptotympana atrata* Fabricius, 1775 (Hemiptera: Cicadidae) in the Korean Peninsula: Insights into Population Structure with Novel Pathogenic Or Symbiotic Candidates

**DOI:** 10.2174/0113892029314148240820082402

**Published:** 2024-08-27

**Authors:** Jeong Sun Park, Jina Kim, Yeha Kim, Ki Hwan Kim, Woori Kwak, Iksoo Kim

**Affiliations:** 1 Department of Applied Biology, College of Agriculture & Life Science, Chonnam National University, Gwangju, 61186, Republic of Korea;; 2 Department of Biotechnology, The Catholic University of Korea, Bucheon, 14662, Republic of Korea;; 3 Gencube Plus, Seoul, 08592, Korea;; 4 Department of Medical and Biological Sciences, The Catholic University of Korea, Bucheon, 14662, Republic of Korea

**Keywords:** Blackish cicada, *Cryptotympana atrata*, hemiptera, whole genome, genetic population, climate-sensitive indicator species

## Abstract

**Background:**

The blackish cicada (*Cryptotympana atrata*) exhibits unique characteristics and is one of the model cicadas found in the Korean Peninsula. It is a species of southern origin, prefers high temperatures, and is listed as a climate-sensitive indicator species in South Korea. Therefore, this species can be utilized to study the impact of climate change on the genetic diversity and structure of populations. However, research on the genome of *C. atrata* is limited.

**Methods:**

We sequenced the genome of an individual collected from South Korea and constructed a draft genome. Additionally, we collected ten specimens from each of the five regions in South Korea and identified single nucleotide variants (SNVs) for population genetic analysis. The sequencing library was constructed using the MGIEasy DNA Library Prep Kit and sequenced using the MGISEQ-2000 platform with 150-bp paired-end reads.

**Results:**

The draft genome of *C. atrata* was approximately 5.0 Gb or 5.2 Gb, making it one of the largest genomes among insects. Population genetic analysis, which was conducted on four populations in South Korea, including both previously distributed and newly expanded regions, showed that Jeju Island, a remote southern island with the highest average temperature, formed an independent genetic group. However, there were no notable genetic differences among the inland populations selected based on varying average temperatures, indicating that the current population genetic composition on the Korean Peninsula is more reflective of biogeographic history rather than climate-induced genetic structures. Additionally, we unexpectedly observed that most individuals of *C. atrata* collected in a specific locality were infected with microbes not commonly found in insects, necessitating further research on the pathogens within *C. atrata*.

**Conclusion:**

This study introduces the draft genome of *C. atrata*, a climate-sensitive indicator species in South Korea. Population analysis results indicate that the current genetic structure of *C. atrata* is driven by biogeographic history rather than just climate. The prevalence of widespread pathogen infections raises concerns about their impact on *C. atrata*. Considering the scarcity of publicly available genomic resources related to the family Cicadidae, this draft genome and population data of *C. atrata* are expected to serve as a valuable resource for various studies utilizing cicada genomes.

## 1 INTRODUCTION

Cicadas are insects belonging to the family Cicadidae in the order Hemiptera. They are characterized by stout bodies, broad heads, clear-membraned wings, and large compound eyes. There are more than 2,000 to 3,000 species of cicadas [1]. They are divided into periodic cicadas, which appear only at certain times, and annual cicadas, which appear every year [2]. Periodical cicadas, such as *Magicicada*, inhabit only North America, while annual cicadas can be observed throughout the world, including Australia, New Zealand, and East Asia [3].

*Cryptotympana atrata* Fabricius, 1775 (Hemiptera: Cicadidae) is an annual cicada found in the northern part of Southeast Asia, extending from Korea to China [4]. This species is mainly found in temperate and tropical regions, and nymphs can hardly survive in cold areas with low temperatures during winter [5]. With a body length of 40 to 44 mm (62 to 69 mm, including wings), it is the largest cicada species inhabiting the Korean peninsula and is a dominant species that has successfully adapted to urban areas [6]. It develops into an adult insect after a larval stage of about 5-6 years and has the characteristic of having all body parts black except for the wings [7]. *Cryptotympana atrata* prefers hot, flat areas over mountainous areas, and it was primarily found in Jeju, a remote island located about 100 km away from the southernmost point of the Korean Peninsula, and the southern region of the Korean Peninsula until the 1980s. However, their habitat is gradually expanding northward due to global warming, urbanization, and the urban heat island phenomenon [8]. In addition, trees preferred by *C. atrata*, such as *Prunus serrulata* (Japanese cherry), *Zelkova serrata* (Japanese zelkova), and *Platanus orientalis* (Oriental plane), are widely used as street trees in landscaping projects [9], facilitating the adaptation of *C. atrata* to environmental changes. As a result, it is being utilized as a national climate change biological indicator species in South Korea [10]. The *C. atrata* of the Korean Peninsula are also known for their calls, which reach noise levels similar to other cicadas in different regions. Their calls are characterized by white noise-type sounds without any melody [11]. Among the various cicada species in the Korean peninsula, *C. atrata* produces the loudest noise, contributing to the tropical night phenomenon in urban areas and night light pollution from sources, such as streetlights. *Cryptotympana atrata* sings not only during the day but also at night [11], using its crying sound as an adaptation to noisy urban environments [12].

Even though *C. atrata* is a major model insect with unique characteristics, research on its genome is limited. Whole genome sequencing (WGS) has emerged as a pivotal tool in biological research in recent years [13], including studies focused on insects. Recent studies in insect population genomics utilizing WGS have significantly contributed to the understanding of genetic diversity, adaptation mechanisms, and evolutionary history of various insect species [14-17]. Therefore, we performed population-level WGS of *C. atrata* in the Korean Peninsula and conducted population genetic analysis to identify the genetic structure of regional *C. atrata* populations. In addition, the possibility of novel microbial pathogenic or symbiotic candidates related to *C. atrata* was investigated through taxonomic analysis of WGS data from specific regions. The draft genome and population-level WGS data for *C. atrata* in this study provide insights into the evolutionary backgrounds for expanding habitats in the Korean Peninsula with novel pathogenic or symbiotic candidates. These resources can be valuable for research not only on *C. atrata* but also on various species within the Cicadidae family.

## 2 MATERIALS AND METHODS

### 2.1 Sampling and Sequencing

**Fig. (1):**
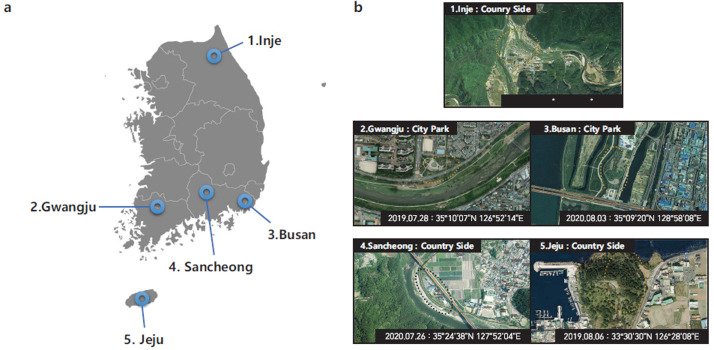
Sampling location of *C. atrata* used in this study. (**a**) Geographical location of 5 regions in the Korean Peninsula. (**b**) Satellite photography with sampling data and GPS coordinates.

Ten specimens were collected from five regions: Inje, Gwangju, Sancheong, Busan, and Jeju (Fig. **1**). The trees where *C. atrata* molts were found in the target area in each region were intensively observed, and the samples were collected when adult *C. atrata* were detected. One or two individuals were collected from each tree. After collection, samples were deposited at –70°C after species identification [18] by one of our morphological specialists. To extract DNA from the collected individuals, one leg from the femur to the tarsal claw was used. The detached leg was washed by spraying with 70% ethanol, and the leg surface was swabbed to remove any potential contamination and remaining ethanol using Kimwipes. This process was repeated twice. Subsequently, to remove the remaining ethanol, the tissue was soaked in a 1.5-mL tube containing 200 μL of TE buffer. The tissue recovered from the TE buffer was swabbed using Kimwipes and dried on Kimwipes. After pulverizing the tissue, DNA was extracted using the Wizard Genomic DNA purification kit (Promega, USA) according to the provided manual. The sequencing library was constructed using the MGIEasy DNA Library Prep Kit following the manufacturer's protocol and sequenced using the MGISEQ-2000 platform with 150-bp paired-end reads.

To obtain mRNA sequencing data for gene prediction, total RNA was extracted from the whole body of a nymph sample collected in Gwangju (CNU16544). After collection, the live sample was soaked in 0.75% sodium chloride, frozen in liquid nitrogen, and stored at –80°C until use. Total RNA was extracted using the RNeasy Micro Kit (Qiagen, Netherlands) according to the manufacturer’s instructions. The mRNA sequencing library was generated using the MGIEasy RNA Library Prep Kit and sequenced using the MGISEQ-2000 platform. Library construction and sequencing were conducted according to the manufacturer’s protocol.

### 2.2 Genome Size Estimation and Draft Genome Assembly

Genome size estimation was performed using the K-mer distribution of the CNU13036 sample from Gwangju, which has high-depth coverage for draft genome assembly. Before analysis, Trimmomatic [19] was used to remove artificial sequences such as sequencing adapters and low-quality bases included in the generated data. The parameters used for Trimmomatic were as follows: ILLUMINACLIP:MGI.fa:2:30:10:2:True LEADING:5 TRAILING:20 MINLEN:125. Two MGI-seq adapter sequences (AAGTCGGAGGCCAAGCGGTCTTAGGAAGACAA, AAGTCGGATCGTAGCCATGTCGTTCTGTGAGCCAAGGAGTTG) were used. Frequencies for 19-mers and 21-mers were calculated using Jellyfish [20] with the parameters “-C -s 10000000 -U 500”. The size of the *C. atrata* genome was estimated using the estimate_genome_size.pl script (https://github.com/josephryan/estimate_genome_size.pl) based on the peak information obtained from Jellyfish. Assembly of the draft genome was performed using Megahit [21] with default parameters. After assembly, BUSCO v5 [22] was used to evaluate the completeness of the constructed draft assembly using the hemiptera_odb10 database with default parameters.

### 2.3 Repeat Element and Gene Modeling

Repeat elements were identified using RepeatMasker [23] to mask repeats present in the genome of *C. atrata*. Before using RepeatMasker, RepeatModeler [24], which includes RECON [25], RepeatScout [26], and TRF [27], was used to create a custom repeat library for the *C. atrata* draft genome using the Dfam repeat library [28] with the parameter “-engine ncbi”. RepeatMasker was performed with the parameter string “-no_is -nolow” using rmblast.

Gene prediction was conducted using RNA-seq data produced by the whole body of *C. atrata* as supporting evidence. The RNA-seq data was filtered using Trimmomatic, and alignment was performed using HISAT2 [29] on the soft-masked genome from RepeatMasker. The resulting bam file was used for gene prediction using Braker2 [30] with default parameters. The protein sequences obtained through gene prediction were compared using diamonds [31] to the NCBI NR database [32], UniProtKB [33], and the genes of the model insect *Drosophila melanogaster* [34] for functional annotation. Matched results were filtered based on an E-value of 1e-5.

### 2.4 Variant Calling and Population Analysis

Reads for each sample filtered using Trimmomatic were aligned to the constructed draft genome using bwa-mem2 [35] with default parameters. PCR duplicates were filtered using the rmdup module of samtools [36], and the genotype for each sample was determined through calling using mpileup of samtools and BCFtools with the parameter string “-mv -Ov”. After the raw variant calling, InDel and multi-allelic loci were removed, and only variants with QV>30 were selected using VCFtools [37] with the parameter string “-minQ 30 -remove-indels -max-missing 1.0 -min-alleles 2 -max-alleles 2”. Annotation of variants was conducted by constructing a custom database for SnpEff [38] based on the constructed gene model using the build module of SnpEff. To confirm the internal population structure using the obtained variant information, the meanQ value was calculated for *K* values of 2, 3, and 4 using fastSTRUCTURE [39] with the parameter string “-full -seed=100”. Subset variants (only missense variants) for general STRUCTURE [40] input were selected based on SnpEff annotation, and calculations were conducted with 50,000 MCMC iterations and 5,000 burn-ins for *K* values of 2, 3, and 4.

### 2.5 Contamination Test and Metagenomic Analysis

Considering the possibility that the low alignment rate of the Busan samples was the result of contamination by microorganisms on the epidermis, we dissected the *C. atrata* and extracted DNA from the internal muscle tissue. Tissue and DNA extraction were performed on the left thorax (LT) and right thorax (RT) muscles of CNU14323, which showed the lowest mapping rate. First, 70% ethanol was sprayed on the CNU14323 specimen to clean the exterior, and the remaining ethanol was removed using Kimwipe. The torso of CNU14323 was dissected using a sterilized knife and tweezers to isolate the muscle tissue, which was then placed in a 1.5-mL tube containing 500 μL of 70% ethanol. After the initial washing, the remaining ethanol was removed using Kimwipe. Subsequently, the tissue was placed in a 1.5-mL tube containing 500 μL TE buffer for final washing, and the excess TE buffer was removed using Kimwipe. The tissue was pulverized to perform DNA extraction using the Wizard Genomic DNA purification kit (Promega, USA) according to the provided manual. NGS data generation using NovaSeq 6000 was performed by following the same procedure as whole genome data generation for the *C. atrata* population. Metaphlan4 [41] with default parameters was used to identify microbial taxa.

## 3 RESULTS

### 3.1 Sample Information and Data Generation

Coordinates, locations, and data produced for each region where sampling was conducted are summarized in Fig. (**1**) and Table **1**. Ten individuals were collected from each of the five regions, and sample CNU13036, collected in the Gwangju region, underwent deep sequencing for genome size estimation and draft genome assembly. For CNU13036, the sequencing data was approximately 164 Gb, while the average size for the remaining samples was approximately 34 Gb, ranging from 30 Gb to 41 Gb. RNA-seq data was approximately 40 Gb.

### 3.2 Genome Size Estimation and Draft Genome of *C. atrata*

The size of the *C. atrata* genome predicted from the constructed K-mer distribution was approximately 5.0 Gb (19-mer) and 5.2 Gb (21-mer), which is similar to the size of the predicted Cicadidae genome reported based on the C-value. Table **2** shows the assembly statistics for the draft genome of *C. atrata*. The draft genome of *C. atrata* generated using Megahit was 4.96 Gb in size, which is similar to the estimated genome size using the 19-mer distribution. The N50 Length of the draft genome assembly was 4,822 bp, the length of the longest contig was 144,147 bp, the average length was approximately 1,136 bp, and the GC content of the assembled genome was 34.91%. Table **3** shows the results of the evaluation of the assembly and gene prediction using BUSCO. In the BUSCO results using hemiptera_odb10, the *C. atrata* draft genome (gene prediction) had 805 (618) complete singles, 29 (44) complete duplicates, 554 (718) fragmented, and 1,122 (1,130) BUSCO genes. In conclusion, the *C. atrata* genome constructed using only short reads was highly fragmented. This fragmentation is likely due to the repetitive sequences that complicate assembly in the large genome of *C. atrata* and the high heterozygosity revealed by *K*-mer distribution, which are difficult to resolve using short reads alone. Nevertheless, given the scarcity of public genome resources for *C. atrata*, this is the first genome assembly that reflects the entire *C. atrata* genome and can be a valuable resource for various Cicadidae genome studies.

### 3.3 Repeat Elements and Gene Model

Before performing gene prediction, a custom repeat library for the *C. atrata* genome was constructed using RepeatModeler, and the repeat elements were identified for the *C. atrata* genome using RepeatMasker. Table **4** shows the identified repeat element information for *C. atrata*. Retrotransposons in the *C. atrata* genome identified through RepeatMasker were approximately 23.88% of the entire genome. Among these, SINE elements accounted for 0.16%; LINE elements, 21.03%; and LTR elements, approximately 2.7%. Most retrotransposons were identified as LINE elements, with RTE/Bov-B (9.7%) and Penelope (4.27%) being the most prevalent elements within this category. DNA transposons accounted for about 7.09% of the *C. atrata* genome, and hobo-Activator (1.68%) and Tc1-IS630-Pogo (3.23%) appeared as the representative DNA transposons. The ratio of unclassified elements, the type of which cannot be determined, was about 29.2%, and total interspersed repeats were about 60.16% of the entire genome.

Gene prediction for the constructed draft genome was performed using Braker2. To increase the accuracy of gene prediction, RNA-seq data from whole-body samples were mapped using HISAT2, resulting in an alignment rate of 87.84%. This indicates that the draft genome of *C. atrata* constructed in this study sufficiently reflects the entire genome. A total of 101,118 genes were predicted in Braker2. The number of predicted genes was quite large, likely due to the draft genome being highly fragmented rather than the actual number of genes in *C. atrata* being so high. For functional annotation of the obtained protein sequences, a diamond search was performed on the protein sequences of the NCBI NR database, UniProtKB, and the *D. melanogaster* gene set proteins. Matches were found for 30,209 (17,899, qcov > 90) in the NR Database, 15,110 in UniProtKB (7,992, qcov > 90), and 15,578 (7,973, qcov > 90) in the *D. melanogaster* gene set. However, after further filtering based on subject coverage above 60, the number of genes meeting the criteria decreased to 2,254 for NR, 660 for UniProtKB, and 689 for the *D. melanogaster* protein set. As expected, gene prediction was also fragmented as the current assembly was constructed only with short reads. Therefore, to enhance the *C. atrata* gene set in the future, the continuity of the genome assembly may be increased by incorporating long-read sequencing platforms such as PacBio and Nanopore.

### 3.4 Regional Population Structure of *C. atrata* in the Korean Peninsula

**Fig. (2):**
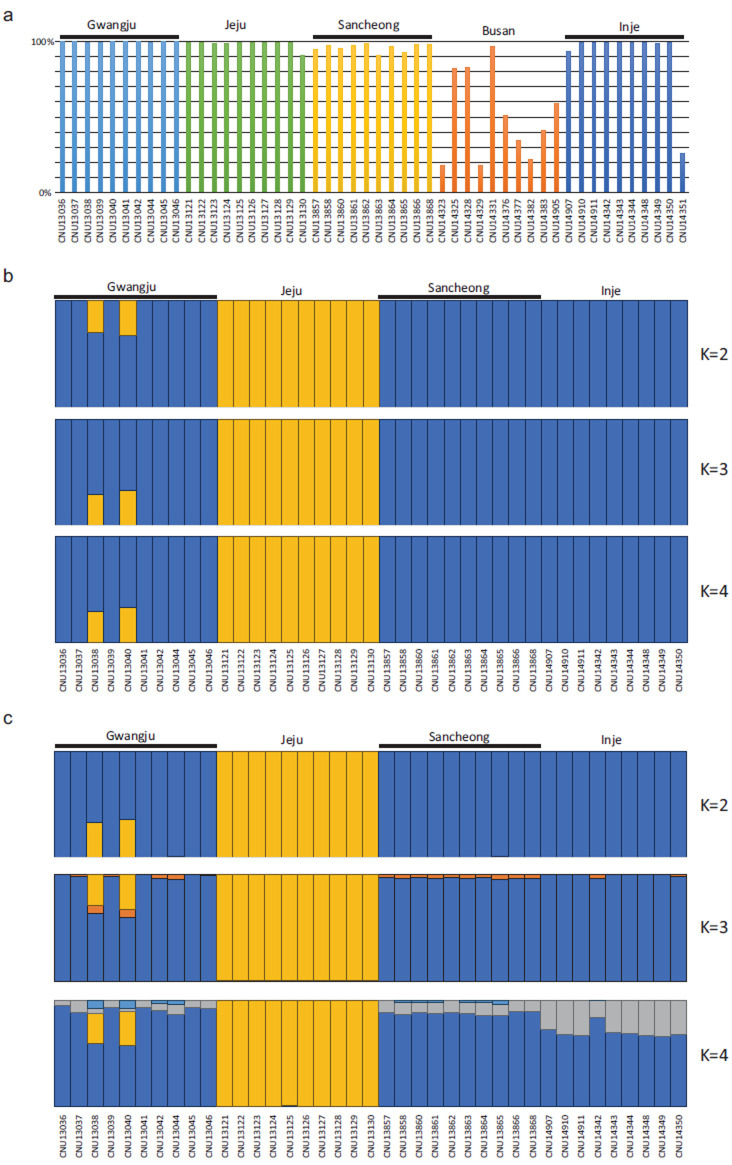
Read alignment rate and genetic structures of *C. atrata* in the Korean peninsula. (**a**) Read alignment rate for each sample using bwa-mem2. (**b**) Population structure analysis using fastSTRUCTURE with 48 million SNVs. (**c**) Population structure analysis using STRUCTURE with 197,558 missense SNVs.

To analyze population structure using whole-genome data, variant information for each individual was obtained. Reads were filtered using Trimmomatic and mapped using BWA-MEM2. Fig. (**2a**) shows the alignment rate for each sample. Among the samples from five regions, those from four regions exhibited a high alignment rate to the constructed draft genome, while samples from the Busan region tended to have a lower mapping rate compared to other regions. In addition, one sample from the Inje region also showed a low mapping rate of 25.75%. Considering the potential impact on the variant calling process, samples with low mapping rates, including those from the Busan area and the Inje area, were excluded from downstream analysis. A total of 106,177,462 single nucleotide variants (SNVs) were initially identified through variant calling, with 48,772,620 variants remaining for the four groups after filtering out multiallelic loci, loci with missing samples, and SNVs with a variant quality score of less than 30 using VCFtools. Fig. (**2b**) shows the population structure of the four regional groups for the *K* values of 2, 3, and 4 analyzed using fastSTRUCTURE with 48,772,620 SNVs. At all *K* values, the Jeju region, which is an island, was clearly differentiated from other regions, while the three inland regions showed no differences in population structure. Although fastSTRUCTURE can analyze population structure using all 48 million SNVs, it lacks resolution when predicting detailed internal population structure. Therefore, STRUCTURE analysis was performed using 197,558 missense SNVs based on SnpEff annotation (Fig. **2c**). In this analysis, the Jeju region was predicted to have a unique internal population structure consistent with fastSTRUCTURE results. While the internal population structure for three regions was shown in more detail compared to fastSTRUCTURE results, it was difficult to discern significant differences among regions in the internal population structure. A small similarity with the Jeju region was observed in some samples from the Gwangju region, which is geographically closest to the Jeju region.

### 3.5 Novel Pathogenic or Endosymbiotic Candidates of *C. atrata*

Even though the sampling and data production processes were consistent across all regions, the data produced in Busan showed a very low BWA-mem2 mapping rate, unlike the other three regions. To investigate the possibility of external contamination affecting the results, additional data production and analysis were conducted. To minimize the potential impact of external microorganism contamination, the CNU14323 sample with the lowest mapping rate in the Busan area was dissected to extract two additional internal pectoral muscle tissues for DNA extraction and shotgun sequencing. The whole genome data consisted of 46,838,137,200 bp for the left thorax (LT) and 48,258,850,800 bp for the right thorax (RT). However, the alignment of the constructed genome resulted in a very low mapping rate of about 3%. Although the sequencing was performed on internal tissues through dissection to eliminate the possibility of external contamination, the mapping rate was still low, and the produced data barely included the genome of *C. atrata*. Even if the sample used was another species similar in appearance to *C. atrata*, a 3% mapping rate was deemed insufficient to be considered WGS data for a related species. Accordingly, to determine its origin, the sequencing data was profiled using Metaphlan4 (Table **5**). The results revealed that the proportions of unclassified sequences were 24.12% and 26.33% for LT and RT, respectively, while the remaining 75% were identified as microorganisms. Twelve microbial species were detected, and the species identified to be present at more than 1% were *Acinetobacter baumannii* (GCF_008632635), *Aeromonas hydrophila* (GCF_016026875), and *Providencia rettgeri* (GCF_003204135). To confirm the presence of these microbial species genomes, mapping coverage was analyzed by aligning reads against the NCBI RefSeq genomes for the three species, confirming high mapping coverage for all three genomes.

## 4 DISCUSSION

While cicadas, including *C. atrata*, are model insects with a plethora of distinctive characteristics, the availability of genome data remains inadequate. The genome size of *C. atrata*, about 5 Gb, is much larger than that of common insects. This increase in genome size is mainly achieved by the expansion of repeat elements. The amount of repeat elements, accounting for about 60% of the total genome, was about 1.5 times greater than that in common mammal genomes. One of the major retrotransposon elements in *C. atrata* was Bov-B, which was initially discovered in the Bovine genome [42] and found to be a major LINE element in two previously reported moth genomes [43, 44]. In terms of SINE elements, similar to other insect genomes, their presence was minimal compared to LINE elements, suggesting that retrotransposons played a key role in shaping the large genome of *C. atrata*. However, during repeat identification using RepeatModeler and RepeatMasker, approximately 50% of the interspersed genome of *C. atrata* was annotated as unclassified. The high proportion of unclassified repeat elements, accounting for about half of all repeat elements, is higher compared to other insect genomes, indicating the likely presence of major lineage-specific repeat elements related to the expansion of the Cicadidae genome. Therefore, additional research on unique repeat elements associated with the expansion and evolution of the Cicadidae genome will be needed in the future. In conclusion, the *C. atrata* draft genome constructed in this study was based solely on MGI short-read data and is highly fragmented. Accordingly, it does not meet the standards of high-quality genome assembly that might be achieved through long-read technologies such as PacBio or Nanopore. Nevertheless, given the scarcity of public genome resources for *C. atrata*, this assembly represents the first depiction of the entire *C. atrata* genome can serve as a valuable resource for various studies on Cicadidae genomes.

Genetic population analysis was conducted to examine if any population structure was present in relation to habitat expansion. The results revealed clear genetic distinctions only between the remote island Jeju and inland populations (Fig. **2b** and **2c**). As typical cicadas, including *C. atrata*, do not fly long distances after reaching adulthood, the observed genetic distinction in Jeju could be attributed to the geographic isolation of the island, which is approximately 100 km away from the nearest southernmost point of the Korean Peninsula. Nevertheless, the slight similarity observed between Jeju and some samples from the Gwangju region could be explained by passive dispersal, such as typhoons that frequently occur in both Jeju and the Korean Peninsula during the summer. In the case of the inland region, the three regions showed no differences in internal population structure, even though the Inje region, which is the northernmost area in the current study, has relatively lower temperatures compared to southern regions such as Gwangju and Sancheong. This suggests that the *C. atrata* population in the Inje region did not expand its habitat through adaptation to lower temperatures, although the rising temperatures in the area due to global warming created a suitable environment for *C. atrata* to thrive. In fact, data from the Korea National Statistical Office (https://kosis.kr/eng/) indicated that the average minimum temperature during the summer in the Inje area increased from 16.9°C to 20.1°C. This temperature was higher than the lowest summer temperatures of 19.4°C and 18.6°C in Gwangju and Sancheong in the 1980s when *C. atrata* mainly inhabited the southern area of the Korean peninsula [12]. The population structure of *C. atrata* in the Korean peninsula, as observed in this study, serves as an example where the impact of climate warming can be confirmed through the genomic characteristics of organisms. This underscores the genetic basis for using *C. atrata* as an indicator species for monitoring climate change due to global warming in the Korean peninsula.

Furthermore, we made an unexpected discovery that the majority of *C. atrata* individuals collected from a specific location were found to be infected with pathogens. Microbial endosymbionts have previously been reported in a variety of insect species [45-47]. In the case of Cicadidae, microbial species such as *Candidatus* Hodgkinia cicadicola are well-known as endosymbionts of seventeen-year cicadas [48]. These microorganisms are known to share the host cell machinery as endosymbionts, and their genomes are reduced in size and fragmented [49]. However, it was confirmed that *C. atrata* does not have these endosymbiotic microorganisms, indicating that endosymbionts may differ depending on the cicada species. In addition, mapping coverage confirmed the presence of genomes of all three major species, showing a different pattern from the genome reduction of *Candidatus* Hodgkinia cicadicola. The three major species identified in the analysis are well-known as major pathogens capable of opportunistic infections, according to previous research. *Acinetobacter baumannii*, which accounts for the largest proportion, is a well-known pathogen with the ability to secure metal nutrients by penetrating host cells and strong antibiotic resistance [50, 51]. *Aeromonas hydrophila* is a pathogen known to mainly infect fish, with the ability to infiltrate and survive within host cells [52, 53]. Lastly, *P. rettgeri* is also recognized as an opportunistic pathogen for various animals, including humans [54], and its infections have been reported to be highly toxic to insects [55]. Considering these known characteristics, it is believed that the identified microbial species, including the three major strains, are likely to be infected pathogens rather than endosymbionts. The fact that this phenomenon was only observed in some regional samples suggests a higher likelihood of opportunistic infections rather than essential endosymbionts for *C. atrata* living on the Korean peninsula. However, recent studies have reported that the genome of microbial endosymbionts can be larger than that of independent external strains [56]. In addition, it was confirmed that the types of endosymbiont microorganisms differ depending on the Cicadidae species, and the pathogenicity of the same microbial species may vary depending on the host [57]. Therefore, further research is needed to confirm whether these microorganisms, which occupy most of the thorax tissue of living cicadas, are simple opportunistic pathogens or endosymbionts.

## CONCLUSION

In this study, we, for the first time, constructed the draft genome of *C. atrata*, which is listed as a climate-sensitive indicator species in South Korea. The *C. atrata* genome is approximately 5 Gb in size, which is much larger than typical for insects. About 60% of it consists of repeat elements, half of which are unclassified, suggesting that the expansion of the *C. atrata* genome may be related to the expansion of lineage-specific repeat elements. Population genetic analysis, conducted to uncover the impact of global warming on range expansion, showed genetic isolation of the population living only on a remote island, supporting the importance of biogeographic history over current climate-induced structures. Unexpectedly, we detected pathogen infections in the majority of individuals collected in a specific locality, raising questions about their impact on *C. atrata*. Further research is essential, especially considering that these pathogens are not well-known in insects. Given the limited availability of public genome resources on Cicadidae, the current *C. atrata* genome could be a valuable resource for genomic studies on cicadas and related groups.

## AUTHORS’ CONTRIBUTIONS

Jeong Sun Park contributed to conceptualization, methodology, investigation, resources, writing – original draft, writing – review and editing. Jina Kim contributed to software, data curation, investigation and validation. Yeha Kim designed the software and helped in visualization. Ki Hwan Kim contributed to methodology and validation. Woori Kwak contributed to conceptualization, data curation; writing – original draft; writing – review and editing. Iksoo Kim contributed to conceptualization, methodology, supervision, project administration, resources; writing – review, and editing.

## Figures and Tables

**Table 1:** Summary of generated whole genome sequencing data for each population.

**Region**	**Sample-name**	**Total Read**	**Total Bases**	**Read Length**	**SRA Accession**
Gwangju	CNU13036	1,097,645,030	164,646,754,500	150-bp paired-end	SRR27295522
	CNU13037	251,788,904	37,768,335,600		SRR27295521
	CNU13038	233,422,396	35,013,359,400		SRR27295510
	CNU13039	215,085,086	32,262,762,900		SRR27295499
	CNU13040	238,333,246	35,749,986,900		SRR27295488
	CNU13041	243,696,792	36,554,518,800		SRR27295477
	CNU13042	237,238,218	35,585,732,700		SRR27295476
	CNU13044	214,295,234	32,144,285,100		SRR27295475
	CNU13045	235,262,952	35,289,442,800		SRR27295474
	CNU13046	220,366,952	33,055,042,800		SRR27295473
Jeju	CNU13121	201,135,868	30,170,380,200		SRR27295520
	CNU13122	244,267,952	36,640,192,800		SRR27295519
	CNU13123	236,574,430	35,486,164,500		SRR27295518
	CNU13124	232,654,806	34,898,220,900		SRR27295517
	CNU13125	248,347,414	37,252,112,100		SRR27295516
	CNU13126	252,527,092	37,879,063,800		SRR27295515
	CNU13127	255,729,714	38,359,457,100		SRR27295514
	CNU13128	221,765,148	33,264,772,200		SRR27295513
	CNU13129	235,098,278	35,264,741,700		SRR27295512
	CNU13130	222,981,680	33,447,252,000		SRR27295511
Sancheong	CNU13857	207,235,604	31,085,340,600		SRR27295509
	CNU13858	238,639,606	35,795,940,900		SRR27295508
	CNU13860	236,841,234	35,526,185,100		SRR27295507
	CNU13861	233,119,020	34,967,853,000		SRR27295506
	CNU13862	235,412,228	35,311,834,200		SRR27295505
	CNU13863	222,878,562	33,431,784,300		SRR27295504
	CNU13864	250,047,780	37,507,167,000		SRR27295503
	CNU13865	263,204,200	39,480,630,000		SRR27295502
	CNU13866	246,879,420	37,031,913,000		SRR27295501
	CNU13868	231,336,240	34,700,436,000		SRR27295500
Busan	CNU14323	243,565,282	36,534,792,300		SRR27295498
	CNU14325	222,063,824	33,309,573,600		SRR27295497
	CNU14328	215,947,988	32,392,198,200		SRR27295496
	CNU14329	227,950,064	34,192,509,600		SRR27295495
	CNU14331	222,857,202	33,428,580,300		SRR27295494
	CNU14376	209,280,698	31,392,104,700		SRR27295493
	CNU14377	222,438,950	33,365,842,500		SRR27295492
	CNU14382	214,056,834	32,108,525,100		SRR27295491
	CNU14383	225,508,110	33,826,216,500		SRR27295490
	CNU14905	232,420,090	34,863,013,500		SRR27295489
Inje	CNU14907	231,859,004	34,778,850,600		SRR27295487
	CNU14910	269,761,366	40,464,204,900		SRR27295486
	CNU14911	211,761,258	31,764,188,700		SRR27295485
	CNU14342	216,451,602	32,467,740,300		SRR27295484
	CNU14343	217,243,516	32,586,527,400		SRR27295483
	CNU14344	241,507,880	36,226,182,000		SRR27295482
	CNU14348	213,287,402	31,993,110,300		SRR27295481
	CNU14349	235,865,844	35,379,876,600		SRR27295480
	CNU14350	266,490,596	39,973,589,400		SRR27295479
	CNU14351	275,101,980	41,265,297,000		SRR27295478

**Table 2:** Summary statistics of draft genome assembly of *C. atrata* using megahit.

Number of contig	4,383,199
Number of As	1,669,948,424
Number of Cs	844,026,147
Number of Gs	845,510,402
Number of Ts	1,623,619,299
Assembly sum	4,983,104,272
GC ratio	34.91
Minimum	200
Maximum	144,147
Average	1,136.86
N50	4,822
N90	320

**Table 3:** BUSCO v5 evaluation result with hemiptera_odb10 for *C. atrata* draft genome.

**-**	**Genome**	**Protein**
Complete single	805 (32.1%)	618 (24.6%)
Complete duplicated	29 (1.2%)	44 (1.8%)
Fragmented	554 (22.1%)	718 (28.6%)
Missing	1,122 (44.6%)	1,130 (45.0%)

**Table 4:** Summary statistics of identified repeat elements in *C. atrata* genome.

**-**	**Number of Elements**	**Length Occupied (bp)**	**Sequence (%)**
Retroelements	4,257,836	1,190,027,574	23.88
SINEs	39,578	7,933,311	0.16
LINEs	3,802,600	1,047,734,399	21.03
LTR elements	415,658	134,359,864	2.70
DNA transposons	1,241,800	353,175,793	7.09
Unclassified	8,871,333	1,454,822,956	29.20
Total interspersed repeats	-	2,998,026,323	60.16
Small RNA	53,757	9,921,811	0.20
Simple repeats	83,588	3,833,809	0.08

**Table 5:** Microbial species identified in dissected thorax tissues using MetaPhlAn 4.

**Species Name**	**LT(%)**	**RT(%)**
Unclassified	24.1189	26.3304
*Acinetobacter baumannii*	64.7684	62.1192
*Aeromonas hydrophila*	4.6926	5.3947
*Providencia rettgeri*	3.7703	3.114
*Raoultella ornithinolytica*	0.8182	0.8501
*Enterobacter hormaechei*	0.7644	0.8213
*Serratia marcescens*	0.5194	0.7712
*Bacillus cereus*	0.3054	0.2825
*Kosakonia cowanii*	0.2113	0.2492
*Enterococcus faecalis*	0.0222	0.0378
*Enterococcus mundtii*	0.0043	0.008
*Klebsiella grimontii*	0.0039	0.0201
*Enterococcus casseliflavus*	0.0000	0.0004

## Data Availability

The generated data and constructed draft genome in this study are available under the NCBI database (PRJNA1028348) and Figshare (https://doi.org/10.6084/m9.figshare.26325127.v1).

## LIST OF ABBREVIATIONS

LT: Left Thorax
RT: Right Thorax
SNVs: Single Nucleotide Variants
